# Predicting consumption of the USDA protein sub-recommendations among adults: a reasoned action approach

**DOI:** 10.34172/hpp.025.44443

**Published:** 2025-12-30

**Authors:** Paul Branscum, Karly Geller

**Affiliations:** Department of Kinesiology, Nutrition and Health, Miami University, Oxford, OH

**Keywords:** Meat, Nuts, Protein, Seafood, Theory of planned behaviour

## Abstract

**Background:**

There is reportedly an under-consumption of protein foods from sub-recommendations within the Dietary Guidelines for Americans (meat, poultry, and eggs, seafood, and nuts, seeds and soy-products). The purpose of this study was to operationalise the Reasoned Action Approach (RAA) to better understand the theory-based factors associated with the consumption of all three protein sub-recommendations.

**Methods:**

A national sample of adults in the United States of America (n=372) were recruited. Protein was evaluated using a modified version of a previously validated survey. RAA variables (intentions, instrumental and experiential attitudes, injunctive and descriptive norms, autonomy, and capacity) were measured for each protein sub-recommendation. Linear regression analyses were used to evaluate determinants of intentions and consumption of protein from each sub-recommendation.

**Results:**

Adults predispositions were significantly higher (*P’*s<0.05) for consuming meats, poultry and eggs [intentions (1.43), instrumental (1.51) and experiential (1.49) attitudes, injunctive (1.23) and descriptive (1.14) norms, autonomy (2.16), and capacity (2.07)], compared to the other groups [seafood (intentions (-0.24), instrumental (0.37) and experiential (0.63) attitudes, injunctive (0.17) and descriptive (-0.46) norms, autonomy (1.73), and capacity (0.94)); nuts, seeds, and soy products (intentions (0.57), instrumental (0.76) and experiential (0.85) attitudes, injunctive (0.47) and descriptive (-0.01) norms, autonomy (1.91), and capacity (1.62))]. A significant amount of the variance was explained for each protein sub-recommendation using the RAA variables[meats, poultry and eggs (intentions adjusted R^2^=0.596/ behaviour adjusted R^2^=0.166); seafood (intentions adjusted R^2^=0.691/ behaviour adjusted R^2^=0.363); nuts, seeds and soy-products (intentions adjusted R^2^=0.582/ behaviour adjusted R^2^=0.390)]; however not all variables were significant.

**Conclusions:**

The RAA appears to be a robust model for explaining why adults under consume protein foods from the USDA sub-recommendations. Future public health education interventions should give particular attention to seafood’s and nuts, seeds, and soy products affordability while maintaining palatability.

## Introduction

 Protein is an essential macronutrient used for structures in the human body, including muscle, tendons, hair, and skin, as well as enzymes and hormones that regulate bodily processes such as the immune system. However, protein consumption has not traditionally been a focus of national nutrition and health efforts as data from NHANES (2011-2014) shows ~97% of children and adults already meet recommendations.^1^ There were no recommendations for protein foods in the first two editions of the Dietary Guidelines for Americans.^2,3^ It was not until the third edition of the DGA^4^ that a protein recommendation was created (also known as the meat and beans group), and consisted of eating 2-3 servings of meats, poultry, fish, dry beans, peas, eggs, and/or nuts. This was largely unchanged until the seventh edition of DGA^5^, when the name of the ‘meat and beans’ group changed to the ‘protein group’, and within the protein group, three additional sub-recommendations were made: one for *meat, poultry, and eggs* (adults should consume ~26 ounces per week); one for *seafood* (adults should consume ~8 ounces per week); and one for *nuts, seeds, and soy products* (adults should consume ~5 ounces per week) [Note: These recommendations are meant for individuals who are not vegetarians or vegans, who are not allergic to seafood, or nuts, and who do not follow special diet plans according to their religion or medical condition].^6^

 Current consumption of protein foods from the three sub-recommendations varies. Approximately 70% of adults consume an adequate amount of meat, poultry, and eggs, but less than half (~45%) meet recommendations for nuts, seeds, and soy products, and only 10% meet recommendations for seafood.^6^ As previously noted, the protein sub-recommendations were not developed from an inadequate- or over-consumption of protein, rather they were designed to prevent an inadequate consumption of essential fatty acids (i.e. the omega-3 fatty acids eicosatetraenoic acid [EPA], and docosahexaenoic acid [DHA]), and to prevent the overconsumption of saturated fats found in meats and eggs.^5-7^ Saturated fat intake has long been associated with cardiovascular disease risk and mortality, however, 77% of adults still consume more than recommended ( ≤ 10% of total calories)^6,8,9^ According to data from NHANES (2017-2020), unprocessed red meats are the highest contributor to saturated fat intake.^10^ Concurrently, data from NHANES (2011-2012) shows only 32% of adults, and 5% of children, consume adequate amounts of omega-3 fatty acids which are rich in seafood, despite being associated with several health benefits, such as reduced inflammation, cardiovascular risk, dementia and cognitive decline, and early neurodevelopment.^11^ Long term studies also show the fibre, types of fats, minerals, and other bioactive components found in nuts and seeds have overall positive health outcomes such as lowering inflammation, and improving cardiovascular risk and cognitive performance.^12^

 Theories and models of behaviour change provide stakeholders a conceptual framework towards reifying psychosocial constructs that can be influenced and changed by small group and community interventions. The Reasoned Action Approach (RAA), along with previous versions of the model such as the Theory of Planned Behaviour, has the distinction of being among the most utilised theories in social and behavioural research. In a meta-analysis, interventions based on the RAA/TPB had on average a weighted effect size (δ^) of 0.50.^13^ The RAA posits behaviours are determined by one’s behavioural intentions (or willingness/motivation) and perceived behavioural control (PBC), which consist of one’s capacity (or ability to perform the behaviour) and autonomy. In turn, behavioural intentions are determined by one’s attitude toward the behaviour which contains cognitive (instrumental attitudes) and affective elements (experiential attitudes), perceived norms concerning the behaviour which contains elements of both injunctive and descriptive norms, and PBC.^14^

 Systematic reviews and meta-analyses show the constructs of the RAA typically have a medium to large positive impact on changing dietary behaviors.^15-17^ The RAA has been used to study the determinants of multiple dietary behaviours, such as the consumption of vegetables,^18-20^ fruit,^18^ sugar-sweetened beverages,^21^ fast-foods,^22^ processed foods,^23^ and dairy products.^24^ The RAA has also been used to study meat consumption and other behaviours related to protein intake. In one study among adults in New Zealand, potential motivators to reduce meat consumption using the RAA was implemented, and attitudes were reported as the only significant predictor of intentions.^25^ Fish and seafood products have also been studies using the RAA as a theoretical framework.^26,27^ However, nuts and seeds alone have not been studied using the RAA, despite the consumption of these foods being included in consuming a plant-based diet.^28^

 While public health programs and initiatives are not needed to promote protein consumption overall, programs that promote higher consumption of seafood and nuts, seeds, and soy products, and moderate consumption of meat, poultry, and eggs, would stand to promote better health outcomes for adults. To date, no study has applied the RAA towards understanding the three sub-recommendations of the USDA’s protein food group, therefore the purpose of this study was to operationalise the RAA to better understand the predisposing, reinforcing, and enabling factors associated with the consumption of all three sub-protein groups. Specific research questions for this study include:

1. Are there differences in theory-based predispositions (instrumental and experiential attitude, injunctive and descriptive norms, capacity and autonomy) towards consuming each protein sub-recommendation (meats, poultry and eggs; seafood; and nuts, seeds and soy products) in the next month?
2. To what extent are the RAA constructs (instrumental and experiential attitude, injunctive and descriptive norms, capacity and autonomy) associated with behavioural intentions towards consuming each protein sub-recommendation (meats, poultry and eggs; seafood; and nuts, seeds and soy products) in the next month?
3. To what extent are behavioural intentions and PBC (capacity and autonomy) associated with consuming each protein sub-recommendation (meats, poultry and eggs; seafood; and nuts, seeds and soy products) in the next month?

## Methods

 All research activities were approved by the sponsoring university’s Institutional Review Board (IRB #03276e). The authors also used the STROBE guidelines for this study. Participants were adults of all races, genders, locations across the United States, socioeconomic statuses, and age groups (18 to 74 years old) who were recruited via Forthright Access (https://www.forthrightaccess.com), a third-party paneling service in May 2023. Forthright conducts its own national recruitment and does not rely on 3rd party advertisers to help control the methods of recruitment and quality of participation of its panelists. Panelists are generally recruited through a diverse set of online and offline advertising channels, however this study only accessed online participants. Forthright also monitors their panels so that inattentive, fraudulent, and poor-quality panelists are not allowed to participate. Potential panelists were sent information about the study and the informed consent form. Those who chose to participate were sent a link to an online survey via Qualtrics and first completed an online consent form. Next, participants were screened out if they reported being a vegetarian or vegan, had a nut/seed allergy, or a seafood allergy.

 Participants were next randomised to one of three surveys, specific to one of the protein sub-recommendations. Participants were given the definitions and descriptions of the protein sub-recommendation for which they were assigned. For example, participants in the nuts, seeds and soy products group were told to consider the behaviour ‘Eating 5 ounces of nuts, seeds and soy products every week for the next month’, and reminded that this was a weekly recommendation that could be done over a few days. Participants were also presented with common examples of foods within each group (i.e. nuts (tree nuts and peanuts), nut butters, seeds (e.g., chia, sesame, and sunflower), and soy which included tofu, tempeh, and products made from soy) and foods that were not included in each group (i.e. any form of meat, poultry, and eggs; any form of seafood; and any other source of protein, like milk, beans, or protein supplements that are not soy-based (e.g. protein bars or powders/shakes)). Finally, participants were given common portion sizes for foods within the group they were assigned (i.e. 1 tablespoon of peanut butter and ½ ounce of nuts or seeds). Survey questions that followed included, protein consumption, and the RAA-based items.

 A modified version of a previously validated food frequency questionnaire was utilised to evaluate protein consumption for each sub-recommendation.^29^ Intake of common food and beverage items containing protein was evaluated, and participants reported how often they consumed items every week (i.e. deli meat). The scale was modified to assure items adequately measured each subgroup. For example, one item (*How many times are you eating red meat, poultry, or fish per week?*) was modified to have separate items for red meat, poultry and pork and dissociate fish from the question. Also, items were added to account for foods not evaluated in the original instrument such as (*How many times are you eating nuts, seeds, and soy products per week? (1 ounce portion)*. To see item weightings, refer to Morin and colleagues.^29^

 Next, the constructs of the RAA were operationalised for each protein sub-recommendation. As Fishbein and Ajzen^14^ note, the first step towards measuring RAA constructs is clearly defining a behaviour to include a *target, *an *action, *a *context *for the action, and a *time *period in which the behaviour is performed is required (i.e. the TACT principle). TACT-specific behaviours were developed to reflect each protein sub-recommendation and included: 1) *Eating 3-4 ounces of meat, poultry, and eggs every day for the next month; *2) *Eating 8 ounces of seafood every week, for the next month; *3) *Eating 5 ounces of nuts, seeds and soy products every week for the next month.*

 Example questions for each scale can be found on Table 1. Items were measured on 7-point semantic differential scales. Behavioural intentions were evaluated using three items for each sub-recommendation. Six total items were used to evaluate instrumental (3-items) and experiential attitudes (3-items), for each sub-recommendation Perceived norms comprised of two normative influences: injunctive, and descriptive norms. For each sub-recommendation three items measured injunctive norms and three items measured descriptive norms. Finally, PBC comprised of two elements: capacity and autonomy. For each sub-recommendation three items evaluated capacity to perform the behaviour, and three items evaluated autonomy over the behaviour.

**Table 1:** Survey Items for Each Reasoned Action Approach Protein Survey

	**How behaviors were framed for each survey. **
Reasoned Action Approach Constructs	-Eating 3-4 ounces of meat, poultry, and eggs every day for the next month. -Eating 8 ounces of seafood every week, for the next month. -Eating 5 ounces of nuts, seeds and soy products every week for the next month.
Intentions	I intend to < …engage in x behavior > ^1^ I plan to < …engage in x behavior > I will < …engage in x behavior >
Attitudes towards a behavior	For me < …engaging in x behavior > is… *--Experiential Attitudes* < 100% Frustrating/100% Enjoyable > < Completely aggravating/Completely satisfying > < Completely unpleasant/Completely pleasant >
	--*Instrumental Attitudes* < Not at all important/Extremely important > < Completely worthless/100% valuable > < Completely too time consuming/Not too time consuming at all >
Perceived norms	--* Injunctive Norm* Most people who are important to me want me to < …engage in x behavior > .^1^ People who are significant to me think it is < Not at all Important/Completely Important > for me to < …engage in behavior x > . Most people whom I respect would < Completely Oppose/Completely Support) me < …engaging in behavior x > .
	--*Descriptive Norm* Most people who are important to me < …engage in behavior x > .^1^ Everyday most people like me < …engage in behavior x > .^1^ How many people similar to yourself < …engage in behavior x > . (None of them/All of them)
Perceived behavioral control	*--Capacity * If I wanted to, I could < …engage in behavior x > .^1^ I have the ability to < …engage in behavior x > . (Definitely Not Able/Definitely Able) To what extent do you see yourself as capable of < …engaging in behavior x > . (Completely Incapable/Completely Capable)
	*--Autonomy* I have (No Control/Complete Control) over whether or not I (…engage in behavior x). < Engaging in behavior x…) is completely up to me.^1^ Whether or not I < …engage in behavior x > is entirely my decision. (Definitely False/Definitely True)

^1^It is scored from Strongly Agree to Strongly Disagree. The other items have the response in the item.

 IBM SPSS Statistics (Statistical Product and Service Solutions, Armonk, NY) version 29 was used for data analyses. Means and standard deviations for each variable are presented on Table 2. All RAA scales were normalised to −3 to + 3 by taking the mean of the items in the scale [i.e. indicating a strong negative attitude (−3) to a strong positive attitude ( + 3)]. In a few rare cases, missing data were handled using the mean replacement method. The internal consistency reliability was also established using Cronbach’s alpha for each RAA construct. For each scale, the following criteria was used to interpret the results: α > 0.8 was deemed *good*; 0.80 > α > 0.7 was deemed *acceptable*; 0.70 > α > 0.6 was deemed *questionable*; 0.60 > α > 0.5 was deemed *poor*; and an α < 0.5 was deemed *unacceptable.*^30^

**Table 2:** Theory-based comparisons by protein sub-recommendation

**Variable**	**Meat group (n=124)** **Mean (SD)**	**Seafood group (n=124)** **Mean (SD)**	**Nuts/Seeds group (n=124)** **Mean (SD)**	***P*** ** value**
Behavioral intentions	1.43 (1.3)^1,2^	-0.24 (2.1)^1,3^	0.57 (1.8)^2,3^	< 0.001
Instrumental attitudes towards the behavior	1.51 (1.3)^1,2^	0.37 (1.7)^1^	0.76 (1.6)^2^	< 0.001
Experiential Attitudes towards the behavior	1.49 (1.5)^1,2^	0.63 (1.9)^1^	0.85 (1.9)^2^	< 0.001
Injunctive norms about the behavior	1.23 (1.4)^1,2^	0.17 (1.7)^1^	0.47 (1.6)^2^	< 0.001
Descriptive norms about the behavior	1.14 (1.4)^1,2^	-0.46 (1.6)^1^	-0.01 (1.5)^1^	< 0.001
Capacity over the behavior	2.07 (1.1)^1^	0.94 (1.9)^1,2^	1.62 (1.5)^2^	< 0.001
Autonomy over the behavior	2.16 (1.1)^1^	1.73 (1.6)^1^	1.91 (1.3)	0.048
Observed range: -3 to + 3 Note: PBC (Perceived Behavioural Control)				
Post Hoc Intentions: Meat/Seafood [*P* = 0.001; *d* = 1.16]; ^2^Meat/Nuts/Seeds [*P* = 0.001; *d* = 0.67]; ^3^Seafood/Nuts/Seeds [*P* = 0.001; *d* = 0.42]. Attitudes: Meat/Seafood [*P* = 0.001; *d* = 0.67]; ^2^Meat/Nuts/Seeds [*P* = 0.002; *d* = 0.48]. Instrumental attitudes: Meat/Seafood [*P* = 0.001; *d* = 0.76]; ^2^Meat/Nuts/Seeds [*P* = 0.001; *d* = 0.52]. Experiential attitudes: Meat/Seafood [*P* = 0.001; *d* = 0.51]; ^2^Meat/Nuts/Seeds [p = 0.015; *d* = 0.38]. Perceived norms: Meat/Seafood [*P* = 0.001; *d* = 0.95]; ^2^Meat/Nuts/Seeds [*P* = 0.001; *d* = 0.71]. Injunctive norms: Meat/Seafood [*P* = 0.001; *d* = 0.68]; ^2^Meat/Nuts/Seeds [*P* = 0.001; *d* = 0.51]. Descriptive norms: Meat/Seafood [*P* = 0.001; *d* = 0.1.07]; ^2^Meat/Nuts/Seeds [p = 0.001; *d* = 0.79]. PBC: Meat/Seafood [*P* = 0.001; *d* = 0.61]; ^2^Nuts/Seeds/Seafood [*P* = 0.038; *d* = 0.29]. Capacity: Meat/Seafood [*P* = 0.001; *d* = 0.75]; ^2^Nuts/Seeds/Seafood [*P* = 0.002; *d* = 0.40]. Autonomy: Meat/Seafood [*P* = 0.043; *d* = 0.32].				

 RAA variables by sub-recommendation were compared across each group by using separate ANOVA analyses, and in cases of significance, post hoc testing was completed to understand significant pairwise differences.

 Linear regression analyses were used to evaluate determinants of intentions and protein sub-recommendation intake for all groups. Instrumental and experiential attitudes, injunctive and descriptive norms, capacity, and autonomy served as independent variables to predict intentions. In turn, intentions, capacity, and autonomy served as independent variables to predict grams of protein sub-recommendation. Assumption testing was also completed to test for outliers, multicollinearity, normality, and homoscedasticity. Using G*Power (Version 3.1.9.6; Düsseldorf, DE), an *a priori* sample size of 98 was determined for each group using the following criteria for a linear multiple regression fixed model: an alpha of 0.05, power of 0.80, 6 independent variables; and a medium effect size (f^2^ = 0.15).^31^

## Results

 Cronbach α scores were used to evaluate the internal consistency reliability of each scale, and all were deemed acceptable [meat/poultry/eggs (intentions: α = 0.93; instrumental attitudes: α = 0.84; experiential attitudes: α = 0.96; injunctive norms: α = 0.87; descriptive norms: α = 0.89; capacity: α = 0.90; autonomy: α = 0.89); seafood (intentions: α = 0.98; instrumental attitudes: α = 0.87; experiential attitudes: α = 0.94; injunctive norms: α = 0.89; descriptive norms: α = 0.91; capacity: α = 0.93; autonomy: α = 0.95); nuts/seeds/soy products (intentions: α = 0.96; instrumental attitudes: α = 0.85; experiential attitudes: α = 0.97; injunctive norms: α = 0.87; descriptive norms: α = 0.88; capacity: α = 0.92; autonomy: α = 0.91)]. Missing data was not an overall problem for any variable ( < 1% of cases for each variable).

 Overall, 372 adults participated in this study (124 participants per group). There were no differences between groups with regards to any demographic variable: age [*P* = 0.186: meat (45.3( + /-16.9) years); seafood 44.6( + /-16.7) years; nuts 48.3( + /-16.7) years], gender [Pearson chi-square (11.192, df = 18) = 0.886: meat (41% women/59% men); seafood (40% women/60% men); nuts (39% women/61% men)], income level [Pearson chi-square (3.049, df = 4) = 0.550: meat (50% low-income/40% middle-/10% high-); seafood (45% low-income/42% middle-/13% high-); nuts (51% low-income/42% middle-/7% high-)] or geographic region within the United States [Pearson chi-square (12.019, df = 6) = 0.062: meat (32% northeast/24% midwest/24% south/20% west); seafood (35% northeast/17% midwest/29% south/19% west); nuts (35% northeast/33% midwest/22% south/10% west)].

Table 2 shows all RAA variables were significantly different across groups. Overall, adults had generally moderate to strong predispositions towards eating foods from the meat/poultry/eggs group, and moderate to neutral predispositions towards eating foods from the seafood and nuts/seeds groups. The RAA constructs were also always significantly higher for meat/poultry/eggs group, and significantly lower for the seafood group.

 Group-specific regression models were used to explore determinants of intentions and protein consumption per sub-recommendation. All variables were confirmed as being normally distributed by using measures of skewness and kurtosis. For all regression models, no issues were found for either outliers (examined using Cook’s distance) or multicollinearity (examined using variance inflation factor). For the first model, intentions were predicted by instrumental and experiential attitudes, injunctive and descriptive norms, capacity and autonomy. As

 observed on Table 3, not all variables entered each model as significant. For the meat/poultry/eggs group instrumental attitudes (β = 0.530; p < 0.001), and injunctive norms (β = .228; *P* < 0.015) explained 59.6% of the variance of intentions. For the seafood group instrumental attitudes (β = 0.374; *P* < 0.001), and descriptive norms (β = 0.417; *P* < 0.001) explained 69.1% of the variance of intentions. Finally, for the nuts/seeds group instrumental attitudes (β = 0.370; *P* < 0.001), injunctive norms (β = 0.265; *P* < 0.013), descriptive norms (β = 0.198; *P* < 0.029), and capacity (β = 0.275; *P* < 0.011) explained 58.2% of the variance of intentions. According to standardised beta coefficients, the strongest predictor of intentions for meat/poultry/eggs and seafood was instrumental attitudes, while descriptive norms was the strongest predictor for seafood.

**Table 3:** Parameter estimates and model prediction for determinants of intentions for Protein Sub-recommendations

	**Adjusted R**^2^	**Standardized coefficients β**	**95% Confidence interval for β**	**t**	***P***
Meat/poultry/eggs participants	0.596				
Instrumental attitudes		0.530	0.290 – 0.745	4.509	< 0.001
Injunctive norms		0.228	0.043 – 0.393	2.462	0.015
Experiential attitudes		-0.133	-0.309 – 0.071	-1.244	0.216
Descriptive norms		0.097	-0.080 – 0.266	1.066	0.289
Capacity		0.177	-0.020 – 0.427	1.800	0.074
Autonomy		0.062	-0.130 – 0.274	0.706	0.482
Seafood participants	0.691				
Instrumental attitudes		0.374	0.222-0.718	3.748	< 0.001
Experiential attitudes		0.027	-0.154 – 0.213	0.322	0.748
Injunctive norms		0.064	-0.129 – 0.289	0.761	0.448
Descriptive norms		0.417	0.351 – 0.742	5.537	< 0.001
Capacity		0.094	-0.056 – 0.262	1.283	0.202
Autonomy		-0.029	-0.200 = 0.124	-0.463	0.644
Nuts/seeds/soy participants	0.582				
Instrumental attitudes		0.370	0.162 – 0.663	3.262	< 0.001
Experiential attitudes		-0.125	-0.310 – 0.072	-1.232	0.221
Injunctive norms		0.265	0.062 – 0.518	2.521	0.013
Descriptive norms		0.198	0.025 – 0.443	2.215	0.029
Capacity		0.275	0.074 – 0.568	2.570	0.011
Autonomy		-0.070	-0.353 – 0.169	-0.698	0.487

 For the second set of regression models, consumption of each protein group was predicted by intentions, capacity, and autonomy (Table 4). For all three groups, intentions [meat/poultry/eggs (β = 0.463; *P* < 0.001); seafood (β = .540; *P* < 0.001); and nuts/seeds (β = 0.622; *P* < 0.001)] was the only significant predictor. Intentions accounted for 16.6% of the variance of meat/poultry/eggs, 36.3% of the variance of seafood, and 39% of the variance of nuts/seeds.

**Table 4:** Parameter estimates and model prediction for determinants of protein sub- recommendations

	**Adjusted R**^2^	**Standardized** **coefficients β**	**95% Confidence** **Interval for β**	**t**	***P***
Meat/poultry/eggs participants	0.166				
Intentions		0.463	1.590 – 4.803	4.528	< 0.001
Capacity		-0.098	-3.312 – 1.610	-0.723	0.471
Autonomy		0.046	-1.641 – 2.779	0.370	0.712
Seafood participants	0.363				
Intentions		0.540	1.236 – 2.362	6.329	< 0.001
Capacity		0.122	-0.279 – 1.165	1.214	0.227
Autonomy		0.014	-0.698 – 0.820	0.160	0.873
Nuts/seeds/soy participants	0.390				
Intentions		0.622	0.404 – 0.701	7.369	< 0.001
Capacity		-0.086	-0.359 – 0.180	-0.655	0.514
Autonomy		0.140	-0.114 – 0.441	1.167	0.245

## Discussion

 In conclusions, the purpose of this study was to operationalise the RAA to better understand the predisposing, reinforcing, and enabling factors associated with the consumption of all three protein sub-recommendations. This was done by comparing the RAA constructs between protein sub-groups (Research Question 1), and using the RAA constructs to predict intentions (Research Question 2) and the consumption of each protein sub-group (Research Question 3). With regards to Research Question 1, it was observed that in general, the predispositions for the meats, poultry and eggs group were generally significantly higher than the other protein groups, and predispositions for the seafood group were the lowest. This likely has to do with the overall accessibility meats, poultry and eggs have, and commonality that these foods appear in typical American dishes. Seafood remains challenging however to add to the American diet. Especially as we observed a negative descriptive norm about seafood, which indicates adults generally don’t believe other adults are consuming seafood either, making the behaviour appear non-normative. With regards to Research Questions 2 and 3, we found some over-arching trends and differences between the protein groups. While behavioural intentions was the primary factor related to the consumption of all protein types, showing motivational interventions are likely to be effective for promoting various protein foods, the primary antecedents driving intentions varied between groups. For the meats, poultry and eggs and the nuts, seeds and soy products groups, instrumental attitudes were the primary antecedent of intentions, while for seafood, descriptive norms were the primary antecedent of intentions. Therefore, future interventions attempting to change the intentions of these protein groups should pay close attention to the types of messages they utilize.

 While previous studies have evaluated protein consumption using theoretical models to our knowledge this is the first-time research has been conducted to explore the USDA sub-recommendations as a framework. In the regression models of this study, the variance explained for intentions to consume all three sub-recommendations ranged from 58.2% to 69.1%. This largely aligns with previous meta-analysis published on the RAA, which shows on average the core RAA constructs predict 58.7% of the variance of intentions.^31^ The same meta-analysis also showed on average intentions, capacity and autonomy account for 30.9% of the variance of health behaviours, which again is similar to our results on seafood (36.3%) and nuts/seeds (39%), but not meat/poultry/eggs which was only 16.6% of the variance. This difference in unobserved variance may stem from additional constructs that were not evaluated in this study, a concept oftentimes referred to as the *sufficiency assumption, *where additional predictors may be useful to enhance behavioural and intentional predictions.^31,32^ For example, other factors identified in previous research that may be useful to understand meat/poultry/eggs consumption include gender,^33^ pro-environmental beliefs,^34^ meat-eater identity,^35^ and determinants of mental health such as depression and anxiety.^36^

 One source of protein not explored in this study was the ‘beans, peas, and lentils’ sub-group (legumes), within the USDA vegetables group. Like the protein food group, the vegetable group also has sub-recommendations that were influenced by recommendations from the National Academy of Medicine.^5,37,38^ Legumes hold a unique position in current USA dietary recommendations because they can be counted as both a vegetable and protein food. Legumes are also a good source of other nutrients, such as fibre, iron and folate, and the consumption of legumes are associated with decreased saturated and total fat intake.^39^ Similar to this study, the ‘beans, peas, and lentils’ sub-recommendation of the vegetables group was evaluated using the RAA/TPB, and results showed that college students had the lowest intentions and attitudes scores for beans, peas, and lentils compared to all of the other vegetables sub-groups (including the dark green, orange and red, starchy and the ‘other’ groups).^40^ In addition, attitudes, perceived norms and PBC predicted 54.6% of the variance of intentions, and intentions and PBC predicted 44.3% of beans, peas, and lentils consumption. It should also be mentioned that this study only evaluated the protein recommendations for individuals who do not consider themselves vegetarians or vegans, for which the USDA gives an alternative set of dietary recommendations (Healthy Vegetarian Eating Pattern). Legumes are heavily emphasized within this eating plan, as a sub-group within both the vegetable and protein groups.

 While there were significant differences in all of the RAA constructs between participants in each sub-recommendation, predispositions to consume meat/poultry/eggs group were the highest, and predispositions to consume seafood were the lowest. This corresponds with national data that shows ~70% of adults consume an adequate amount of meat, poultry, and eggs, while only 10% meet recommendations for seafood.^6^ Previous reports have cited cost, taste, health and nutritional beliefs, habits, the availability of seafood and cooking skills as common barriers for seafood consumption.^41^ Future health education interventions should therefore give particular attention to seafood’s affordability and teach how to cook seafood dishes so they are more palatable.

 Results from this study give notable insight into future plans for designing and delivering interventions to shape healthy protein consumption. As Pryor^42^ notes, there are three primary ways of influencing attitudes and norms, which were collectively the strongest predictors for all three protein sub-groups. First, interventions can find ways to strengthen beliefs in behavioural outcomes (for attitudes) or referents (for norms) that are already favourably evaluated. This typically involves reinforcing beliefs adults already have about eating each protein sub-recommendation (e.g. there are health benefits from eating seafood, and nuts/seeds). Next, interventions can find ways to reduce the strength of beliefs in behavioural outcomes or referents that are currently unfavourably evaluated. This involves rectifying myths adults have about protein sub-recommendation (e.g. finding less expensive ways to consume seafood and nuts/seeds when it is perceived too costly). Finally, interventions can teach adults new information about behavioural outcomes or referents that supports consuming the protein sub-recommendation. This could include teaching adults about the specific omega-3 fatty acid found in seafood, and the health benefits associated with their consumption. Also, nuts and seeds are also often viewed as high-fat foods, and should be avoided to prevent weight gain. However, teaching that the fats contained in nuts/seeds are largely from unsaturated fats could be helpful, especially since results from multiple randomised controlled trials have shown their consumption is in fact beneficial for weight control, and preventing long term weight gain.^43^

 There are notable strengths and limitations of this study that should be addressed. One strength to this study was the comprehensive way in which protein intake was evaluated. While protein within itself is a highly studied macronutrient, rarely, if ever, do studies attempt to understand the three sub-recommendations of the USDA’s protein food groups at the same time. Another strength lies within the strong theoretical foundations of this study. The use of the RAA provided a robust framework to explain dietary behaviors, aligning with prior meta-analyses.^31^ Finally, a strength of this study is the national sampling of adults used in order to get a broad perspective about these behaviours. Despite these strengths, there were some limitations that should be addressed. First, given the nature of evaluating psychosocial variables, all data were self-report, therefore participants may not have been honest, or wanted to portray themselves in a more favourable way given the sensitive nature of this study. Second, even though this was a national sampling of adults in the United States, due to constraints it was not a random sampling of adults, which introduces the possibility of selection bias, limiting generalizability. All data were also collected by self-report, which could introduce self-report bias and social desirability. The final limitation was the data presented in this study was cross-sectional, which limits the ability to make claims of causal relationships.

## Conclusion

 These findings underscore the RAA’s utility in designing targeted dietary interventions. Key findings revealed that protein foods from the meat/poultry/eggs group were most favored by respondents, and were driven by instrumental attitudes and injunctive norms. In turn, protein foods from the seafood group were least favored, with descriptive norms as the primary predictor. Protein foods from the nuts/seeds/soy group were influenced by instrumental attitudes, norms, and perceived capacity. Interventions to promote healthy protein consumption should prioritize affordability and palatability for seafood and nuts/seeds, while leveraging motivational messaging tailored to each subgroup.

## Competing Interests

 All named authors have no conflict of interest to disclose, financial or otherwise.

## Ethical Approval

 All procedures performed in studies involving human participants were in accordance with the ethical standards of the institutional and/or national research committee and with the 1964 Helsinki declaration and its later amendments or comparable ethical standards. All research activities were approved by Miami University’s Institutional Review Board (IRB #03276e).
